# Midlife health in Britain and the United States: a comparison of two nationally representative cohorts

**DOI:** 10.1093/ije/dyae127

**Published:** 2024-10-03

**Authors:** Charis Bridger Staatz, Iliya Gutin, Andrea Tilstra, Laura Gimeno, Bettina Moltrecht, Dario Moreno-Agostino, Vanessa Moulton, Martina K Narayanan, Jennifer B Dowd, Lauren Gaydosh, George B Ploubidis

**Affiliations:** Centre for Longitudinal Studies, University College London, London, UK; Department of Sociology, University of Texas at Austin, Austin, TX, USA; The Maxwell School for Citizenship and Public Affairs, Syracuse University, Syracuse, NY, USA; Leverhulme Centre for Demographic Science, Nuffield College, and Nuffield Department of Population Health, University of Oxford, Oxford, UK; Centre for Longitudinal Studies, University College London, London, UK; Centre for Longitudinal Studies, University College London, London, UK; Centre for Longitudinal Studies, University College London, London, UK; ESRC Centre for Society and Mental Health, King’s College London, London, UK; Centre for Longitudinal Studies, University College London, London, UK; Centre for Longitudinal Studies, University College London, London, UK; Leverhulme Centre for Demographic Science, Nuffield College, and Nuffield Department of Population Health, University of Oxford, Oxford, UK; Department of Sociology, University of Texas at Austin, Austin, TX, USA; Department of Sociology, The University of North Carolina at Chapel Hill, North Carolina, USA; Centre for Longitudinal Studies, University College London, London, UK

**Keywords:** Biomarkers, cardiometabolic health, cross-country comparison, mid-life, harmonization, socioeconomic position, inequalities

## Abstract

**Background:**

Older adults in the USA have worse health and wider socioeconomic inequalities in health compared with those in Britain. Less is known about how health in the two countries compares in mid-life, a time of emerging health decline, including inequalities in health.

**Methods:**

We compare measures of current regular smoking status, obesity, self-rated health, cholesterol, blood pressure and glycated haemoglobin using population-weighted modified Poisson regression in the 1970 British Cohort Study (BCS70) in Britain (*N* = 9665) and the National Longitudinal Study of Adolescent to Adult Health (Add Health) in the USA (*N* = 12 300), when cohort members were aged 34–46 and 33–43, respectively. We test whether associations vary by early- and mid-life socioeconomic position.

**Results:**

US adults had higher levels of obesity, high blood pressure and high cholesterol. Prevalence of poor self-rated health and current regular smoking was worse in Britain. We found smaller socioeconomic inequalities in mid-life health in Britain compared with the USA. For some outcomes (e.g. smoking), the most socioeconomically advantaged group in the USA was healthier than the equivalent group in Britain. For other outcomes (hypertension and cholesterol), the most advantaged US group fared equal to or worse than the most disadvantaged groups in Britain.

**Conclusions:**

US adults have worse cardiometabolic health than British counterparts, even in early mid-life. The smaller socioeconomic inequalities and better overall health in Britain may reflect differences in access to health care, welfare systems or other environmental risk factors.

Key Messages

- We set out to understand differences in mid-life health between Britain and the USA, and differences in the extent of socioeconomic inequalities in health between the two countries.
- Middle-aged adults in the USA have worse cardiometabolic health than their British counterparts, although British adults are more likely to engage in unhealthy behaviours; however, socioeconomic inequalities in both cardiometabolic health and health behaviours are typically wider in the USA, such that the most advantaged groups in the USA often have similar or worse health than the most disadvantaged groups in Britian.
- These findings, along with previously published evidence, have implications for policy and practice, as they suggest sociopolitical differences between the two countries that may drive different health profiles. Systematic differences between Britain and the USA in terms of health care and welfare provisions may drive both worse health and wider inequalities in the USA.

## Background

International comparisons document worse health in the USA compared with England.^1–5^ Older US adults are more likely to self-report having diabetes, hypertension, heart disease and other chronic conditions.^2^ They also have a higher average body mass index (BMI) and prevalence of extreme obesity.^4^ However, older English adults exhibit worse health behaviours, including the co-occurrence of smoking, alcohol consumption and sedentary activity, and are less likely to present with no behavioural risk factors.^1^

Previous USA-England comparisons have primarily focused on ages 50+ (late mid-life and older ages), based on harmonized international surveys of ageing,^6^ and mid-life (ages 30–60), and particularly younger mid-life (ages 30–40), is often overlooked.^7^ Yet, there is growing recognition of mid-life’s importance in setting the stage for healthy ageing, marking the start of physical and functional decline.^8^ In contrast to USA-England comparisons at older ages, the limited evidence at younger ages is more equivocal. One comparison finds similar patterns of worse cardiometabolic health but better health behaviours in the USA at ages 35–54.^5^ Conversely, a more recent study examining adults born between 1965 and 1980 documents higher prevalence of hypertension and dyslipidaemia among English adults and comparable prevalence of smoking and diabetes.^9^^,^^10^

Moreover, these mid-life declines in health likely exhibit social gradients. Studies comparing England and the USA document wealth, income and education inequalities across multiple chronic conditions for older adults,^2^^,^^3^ with some evidence of similar gradients in mid-life.^5^^,^^11^ In both countries, behavioural risk factors explain some of these gradients, but a sizeable proportion remains unaccounted for—indicative of the multiple mechanisms through which social disadvantages operate.^3^^,^^5^ Indeed, health inequalities originate even earlier in life, as multiple studies of British and US adults show associations between early life socioeconomic position (SEP) and adult health.^12^^,^^13^

Critically, international comparisons provide the opportunity to identify contextual drivers of population health,^14–16^ with prior work finding smaller health inequalities in countries with higher national incomes, more social transfers and greater investments in health care and policy.^14^ Observed differences between the USA and England have been attributed to the cost of health care,^2^^,^^5^ which is free at the point of access in the UK (England, Scotland, Wales and Northern Ireland), differences in income benefit systems^2^^,^^3^ and the quality of local environments and neighbourhoods.^17^ These contextual determinants likely vary throughout the life course, as does the corresponding level of welfare provisions between countries (e.g. retirement benefits vs childhood welfare).

Thus, we build on previous work and compare behavioural risk factors and biomarkers of health in early mid-life from two nationally representative cohorts, the National Longitudinal Study of Adolescent to Adult Health (Add Health) in the USA and the 1970 British Cohort study (BCS70) in Britain (England, Wales and Scotland). We also consider cross-country differences in health inequalities across different measures of SEP. Specifically, we hypothesize that the US cohort will generally have worse health than their British peers, and that US SEP inequalities will be larger.

## Methods

### Datasets

BCS70 is an ongoing nationally representative birth cohort of ∼17 000 individuals born in 1970 in Britain.^18^ The 10th follow-up (or sweep), in 2016, collected multiple biomedical measures, including blood samples. The current analysis uses data from Sweeps 8 to 10, when cohort members were aged 34 (*N *= 9665), 42 (*N* = 9841) and 46–48 (*N* = 8851).

Add Health is a nationally representative cohort of ∼20 000 individuals in the USA enrolled in grades 7–12 (ages 12–18) in 1994–95.^19^ The fifth follow-up (or wave) occurred in 2016–19 (ages 33–43; *N *= 12 300). Biomedical measures were collected on a subsample of these Wave V participants (*N *= 5381).

### Variables

Our outcome variables were: current regular smoking; BMI; self-rated health; total cholesterol-to-high-density lipoprotein (HDL) ratio; blood pressure; and blood sugar level (glycated haemoglobin [HbA1c], a marker for diabetes). In BCS70, smoking status was measured at age 34, self-rated health at age 42 and all remaining measures at ages 46–48. For Add Health, all measures were taken from Wave V. Outcomes were converted to binary variables using cut-offs shown in Supplementary Materials S1 (available as Supplementary data at *IJE* online).

For chronic diseases (e.g. diabetes) measured through biomarkers (e.g. HbA1c), we distinguish between the biomarker alone, and any indication of the disease (e.g. ‘any diabetes’) based on medication usage for the specific conditions. For obesity, we augment measured height and weight with self-reported height and weight (though we look at measured BMI, exclusively, in supplementary analyses). Full details of the harmonization are shown in Supplementary Material S2 (available as Supplementary data at *IJE* online).

Three measures of SEP were used: parental education (Add Health ages 11–19, BCS70 age 16), own education and household income [Add Health ages 33–43 and BCS70 ages 34 and 42 (dependent on the outcome)]. Parental education was grouped as: (i) neither parent has a university bachelor’s degree; or (ii) at least one parent has a degree. Own education was also grouped based on completing a bachelor’s degree; household income was classified into approximate quintiles. For further details see Supplementary Material S2 (available as Supplementary data at *IJE* online).

BCS70 is largely racially/ethnically homogeneous, with most of the cohort born to UK or European parents (93%) and therefore likely White (see Supplementary Material S2, available as Supplementary data at *IJE* online). In Add Health, race/ethnicity was measured at Wave I; the primary analysis was restricted to non-Hispanic White adults to maximize comparability with BCS70.

In BCS70, the ‘age 46’ biomedical sweep took place across 3 years (ages 46–48); age in Add Health was calculated based on interview and birth date. In the remaining BCS70 sweeps, age was included as a dummy variable (i.e. 34 and 42). Sex assigned at birth was measured in the first sweep in BCS70 and at Wave I in Add Health.

### Statistical analysis

Modified Poisson regression analysis was used to obtain relative risk estimates [risk ratios (RR)]] and corresponding 95% confidence intervals (CI). In Model 1, the independent variable was a dummy for country (Britain or USA) controlled for age. In the sensitivity analysis, the full, racially/ethnically heterogeneous Add Health sample was used. All analyses were conducted on pooled and sex-stratified samples.

Model 2 examined country moderation in the associations between SEP and health, including interaction terms between country and SEP in separate models (one each for parent education, respondent education and household income). For interpretation, RR estimates are presented as adjusted predicted marginal estimates of prevalence, estimated at the observed values of covariates (further details are provided in Supplementary Material S3, available as Supplementary data at *IJE* online). A Wald test indicated whether SEP differences were significant between countries. For household income, the difference between the bottom, middle and top quintiles relative to the second quintile was tested for significance, controlled for household size.

Model 3 examined the relationship between childhood SEP and adult health after controlling for adult education and household income. By adjusting for SEP in adulthood, we conducted an informal mediation analysis, assessing the direct effect of early-life SEP on mid-life health (not mediated by mid-life education and income).

Models 2 and 3 were stratified by sex in supplementary analyses.

Sensitivity analyses compared models using clinically measured obesity in Add Health with the self-report supplemented measure used in the main analysis. We also explored harmonized measures of heavy drinking, based on UK and country specific guidelines, presented in Supplementary material only (available at *IJE* online).

Finally, Add Health uses a complex, stratified sampling strategy,^19^ thus maintaining the national representativeness of the data. Additional survey weights account for non-representativeness among adults providing biomarker samples.^20^ To maximize cross-sample comparability, non-response weights were developed in BCS70. The development of weights and use of a complex survey design are described in Supplementary Material S4 and S5 (available as Supplementary data at *IJE* online). We further detail analytical sample sizes in Supplementary Material S6 (available as Supplementary data at *IJE* online).

## Results

Table 1 shows the weighted proportion of outcomes and covariates in the analytical samples (unweighted distribution in Supplementary Material S7, available as Supplementary data at *IJE* online). US adults had a higher prevalence of obesity and high blood pressure and cholesterol; conversely, British adults had higher prevalence of poor self-rated health and current smoking. University degree attainment was higher among US respondents’ parents (36% vs 21%); respondent university completion was similar (40% versus 36%).

**Table 1.** Weighted distribution of outcomes and covariates in BCS70 and Add Health in analytical sample (non-Hispanic White only)

	BCS70	Add Health
Smoking status		
Regular smoker	27.9%	21.4%
Non-smoker	72.1%	78.6%
Self-rated health		
Poor/fair health	18.3%	12.1%
Good/excellent health	81.7%	87.9%
Obesity		
Obese	34.5%	40.4%
Not obese	65.5%	59.6%
High blood pressure (biomarker)		
Hypertension	19.0%	22.5%
Normal	81.0%	77.5%
High cholesterol (biomarker)		
Unhealthy	7.63%	10.7%
Healthy	92.4%	89.3%
High HbA1c (biomarker)		
Diabetes	5.96%	4.41%
No diabetes	94.0%	95.6%
Any hypertension		
Yes	19.3%	30.4%
No	80.7%	69.6%
Any high cholesterol		
Yes	9.67%	15.3%
No	90.3%	84.7%
Any diabetes		
Yes	7.27%	8.14%
No	92.7%	91.9%
Sex		
Male	55.3%	50.4%
Female	44.7%	49.6%
Parental education level		
Neither parent has a degree	79.3%	64.2%
At least one parent has a degree	20.7%	35.8%
Own education level (Add Health Wave V, BCS70 Sweep 9)		
No university degree	64.5%	60.3%
Degree-level educated	35.5%	39.7%
Own education level (BCS70 Sweep 8 only)		
No university degree	63.7%	–
Degree-level educated	36.3%	–
Own income (Add Health Wave V, BCS70 Sweep 9)		
Lowest income quintile	25.4%	17.7%
Second quintile	20.6%	24.3%
Middle quintile	18.4%	17.4%
Fourth quintile	17.9%	22.0%
Highest income quintile	17.6%	18.6%
Own income (BCS70 Sweep 8 only)		
Lowest income quintile	22.3%	–
Second quintile	20.9%	–
Middle quintile	19.4%	–
Fourth quintile	18.4%	–
Highest income quintile	18.9%	–
Age, mean (SD)^a^		
Wave V (Add Health only)	–	37.4 (1.78)
Sweep 10 (BCS70 only)	46.8 (0.77)	–
Sweep 9 (BCS70 only)^a^	42	–
Sweep 8 (BCS70 only)^a^	34	–

For all outcomes, the values represent weighted proportions (%), apart from age which represents mean and SD.

Add Health, The National Longitudinal Study of Adolescent to Adult Health; BCS70, 1970 British Cohort Study; HbA1c, glycated haemoglobin; SD, standard deviation.

a Age at Sweeps 9 and 8 in BCS70 is included as a dummy variable, for the respective age in years at interview. Therefore, standard deviation for these ages is equal to 0, as all cohort members are allocated the same age in years.

**Figure 1.**
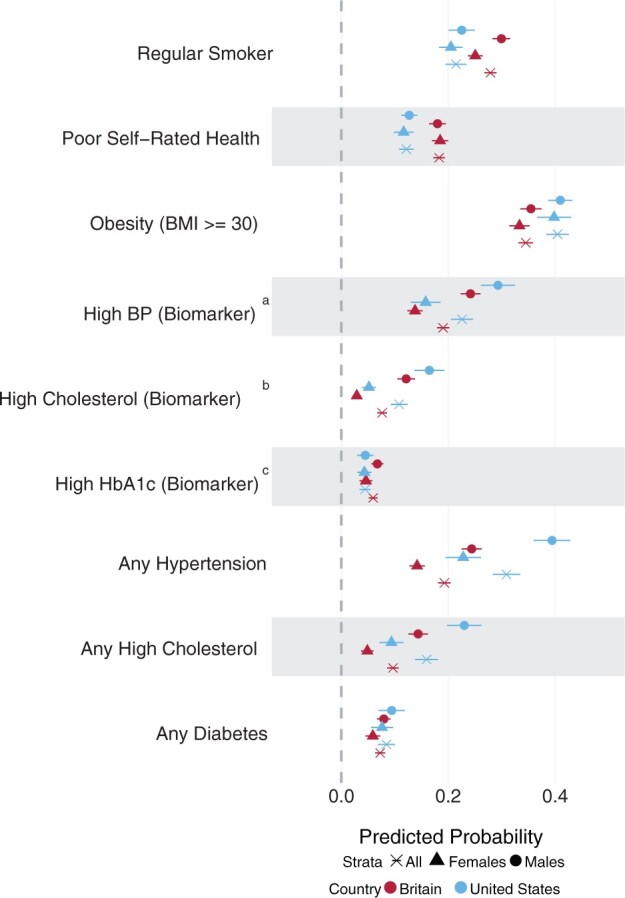
Predicted probabilities from modified Poisson regression, comparing health indicators between Britain and the USA by sex (Model 1). ^a^BP is blood pressure. ^b^High cholesterol is measured by the ratio of total cholesterol (TC) to high-density lipoprotein (HDL). ^c^HbA1c is glycated haemoglobin (blood sugar levels). Outcomes labelled ‘any’ refer to biomarkers that have been supplemented with medication use, therefore indicating a positive diagnosis of diseases. BMI, body mass index; TC, total cholesterol; HDL, high-density lipoprotein; BP, blood pressure; HbA1c, haemoglobin A1c (glycated haemoglobin)

Results from Model 1 show US adults generally had worse cardiometabolic health (Figure 1, Table 2). They were more likely to have high blood pressure and cholesterol, before and after accounting for medication use [any hypertension: 0.309 (95%CI: 0.284, 0.335) vs 0.193 (0.181, 0.204)]; any high cholesterol: [0.159 (0.138, 0.181) vs 0.097 (0.086, 0.107)] and more likely to have obesity [0.405 (0.384, 0.426) vs 0.345 (0.332, 0.358)]. However, British adults were more likely to be current smokers [0.279 (0.268, 0.290) vs 0.214 (0.195, 0.234)] and report poor self-rated health [0.183 (0.172, 0.194) vs 0.122 (0.108, 0.136)].

**Table 2.** Marginal estimates from modified Poisson regression for Model 1, examining country differences in health outcomes: overall and sex stratified

		Britain				USA				
		*n*	Estimate	Lower 95% CI	Upper 95% CI	*n*	Estimate	Lower 95% CI	Upper 95% CI	***P* difference** ^a^
Smoking	All	9634	0.279	0.268	0.290	6675	0.214	0.195	0.234	<0.0001
	Male	4258	0.299	0.283	0.316	2998	0.225	0.201	0.250	<0.0001
	Female	5376	0.251	0.237	0.264	3677	0.205	0.183	0.227	0.0006
Poor self-rated health	All	9798	0.183	0.172	0.194	6717	0.122	0.108	0.136	<0.0001
	Male	4341	0.180	0.164	0.196	3018	0.127	0.112	0.142	<0.0001
	Female	5457	0.185	0.170	0.200	3699	0.117	0.098	0.135	<0.0001
Obesity (BMI ≥30 kg/m^2^)	All	8494	0.345	0.332	0.358	6690	0.405	0.384	0.426	<0.0001
	Male	3790	0.355	0.335	0.375	3000	0.410	0.387	0.432	0.0003
	Female	4704	0.333	0.314	0.352	3690	0.398	0.366	0.430	0.0006
High blood pressure (biomarker)	All	7529	0.190	0.179	0.202	3133	0.226	0.205	0.247	0.0035
	Male	3351	0.242	0.223	0.260	1294	0.293	0.261	0.325	0.0066
	Female	4178	0.138	0.124	0.152	1839	0.158	0.130	0.186	0.2090
High cholesterol (biomarker)	All	6036	0.076	0.067	0.086	2825	0.108	0.092	0.124	0.0006
	Male	2712	0.121	0.105	0.138	1158	0.165	0.136	0.193	0.0094
	Female	3324	0.029	0.021	0.037	1667	0.052	0.039	0.064	0.0028
High HbA1c (biomarker)	All	5996	0.060	0.051	0.068	2786	0.044	0.034	0.055	0.0240
	Male	2705	0.067	0.056	0.079	1145	0.045	0.029	0.060	0.0201
	Female	3291	0.046	0.034	0.058	1641	0.043	0.030	0.056	0.7500
Any hypertension	All	7546	0.193	0.181	0.204	3457	0.309	0.284	0.335	<0.0001
	Male	3357	0.244	0.225	0.263	1484	0.394	0.360	0.429	<0.0001
	Female	4189	0.142	0.127	0.156	1973	0.228	0.195	0.261	<0.0001
Any high cholesterol^b^	All	6127	0.097	0.086	0.107	2974	0.159	0.138	0.181	<0.0001
	Male	2759	0.144	0.125	0.162	1245	0.230	0.198	0.262	<0.0001
	Female	3368	0.049	0.037	0.060	1729	0.094	0.071	0.116	0.0005
Any diabetes	All	6057	0.073	0.063	0.082	2881	0.084	0.068	0.100	0.2100
	Male	2732	0.080	0.067	0.092	1193	0.094	0.069	0.119	0.3006
	Female	3325	0.059	0.045	0.073	1688	0.076	0.055	0.097	0.1700

Results presented are for Model 1, exploring country differences in health outcomes between the UK (1970 British Cohort Study) and USA [National Longitudinal Study of Adolescent to Adult Health (Add Health)]. Outcomes labelled ‘any’ refer to biomarkers that have been supplemented with medication use, therefore indicating a positive diagnosis of diseases.

BMI, body mass index; CI, confidence interval; HbA1c, glycated haemoglobin.

a *P* value is for Wald test, indicating a statistical difference between countries.

b High cholesterol measured by total cholesterol to high-density lipoprotein ratio.

In both cohorts, men were more likely to have high blood pressure and cholesterol than women. British men were also more likely to be current regular smokers. The male health disadvantage was greater for hypertension and high cholesterol in the USA, whereas the male-female gap in current smoking was greater in Britain.

**Figure 2.**
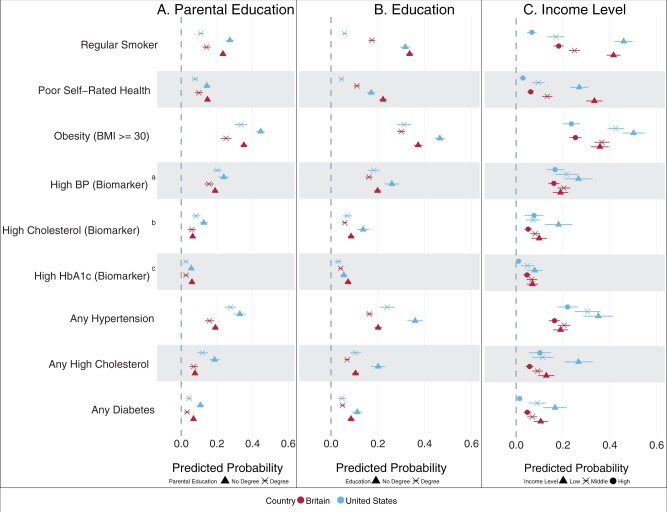
Predicted probabilities from modified Poisson regression showing socioeconomic inequalities in mid-life health between Britain and the USA (Model 2a, b and c). Measures of SEP are parental education (Model 2a), the cohort’s own education level (Model 2b) and household income quintiles (Model 2c, only the first, third and fifth quintiles presented in the figure). ^a^BP is blood pressure. ^b^High cholesterol is measured by the ratio of total cholesterol (TC) to high-density lipoprotein (HDL). ^c^HbA1c is glycated haemoglobin (blood sugar levels). Outcomes labelled ‘any’ refer to biomarkers that have been supplemented with medication use, therefore indicating a positive diagnosis of diseases. BMI, body mass index; TC, total cholesterol; HDL, high-density lipoprotein; BP, blood pressure; HbA1c, haemoglobin A1c (glycated haemoglobin); SEP, socioeconomic position

As seen in Model 2, socioeconomic inequalities in mid-life health were greater for adult SEP compared with childhood SEP (Figure 2; Supplementary Material S8, available as Supplementary data at *IJE* online). Predicted probabilities of current smoking and reporting poor self-rated health were higher for lower-educated and lower-income adults.

In both cohorts there was a small SEP gradient in hypertension and cholesterol, mostly for respondents’ education. In Britain, prevalence of obesity was similar between middle- and low-income groups, whereas the highest-income quintile exhibited a significantly lower level of obesity compared with the other groups; by contrast, in the USA there was a clear income gradient across the distribution [lowest: 0.501 (0.454, 0.549), middle: 0.425 (0.390, 0.459), highest: 0.236 (0.199, 0.273)]. Both countries had educational gradients, especially for respondents’ education. Results were similar in sex-stratified models (Supplementary Material S9, available as Supplementary data at *IJE* online).

For some outcomes, such as current smoking, the most socioeconomically advantaged US adults were healthier than their British peers and the most disadvantaged fared worse, resulting in wider inequalities in the USA [smoking: USA, highest income: 0.067 (0.046, 0.087); Britain, highest income: 0.182 (0.160, 0.203); USA, lowest income: 0.459 (0.421, 0.497); Britain, lowest income: 0.416 (0.387, 0.445)]. Though less clear, we observed a similar pattern for ‘any’ diabetes.

For other outcomes, such as obesity, more advantaged SEP groups in both cohorts had similar probabilities, but higher obesity levels among socioeconomically disadvantaged US adults led to wider inequalities. Conversely, wider inequalities in self-rated health were the result of better self-rated health among advantaged US adults, despite similar poor health among socioeconomically disadvantaged adults in both cohorts. For some outcomes, such as hypertension and cholesterol, more socioeconomically advantaged US adults had similar or worse health than socioeconomically disadvantaged British adults, particularly as measured by parental or adult education [Figure 2, Panel B, ‘Any’ hypertension: USA, degree: 0.240 (0.209, 0.272); Britain, no degree: 0.164 (0.149, 0.179)]. This pattern was also observed for obesity across parental SEP.

**Figure 3.**
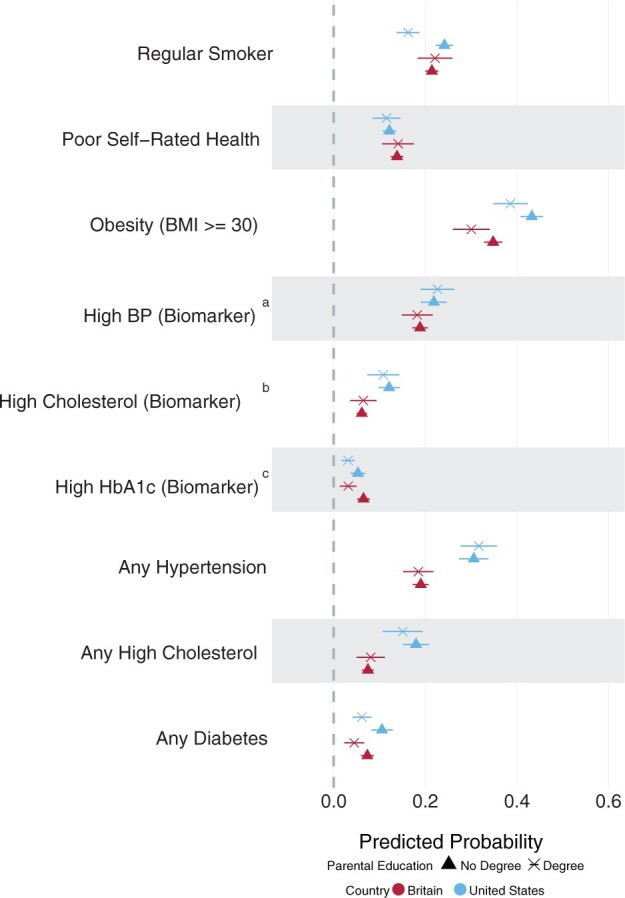
Predicted probabilities from modified Poisson regression of associations with parental education, adjusted for cohort members’ own socioeconomic position (education level and household income) in adulthood (Model 3). ^a^BP is blood pressure. ^b^High cholesterol is measured by the ratio of total cholesterol (TC) to high-density lipoprotein (HDL). ^c^HbA1c is glycated haemoglobin (blood sugar levels). Outcomes labelled ‘any’ refer to biomarkers that have been supplemented with medication use, therefore indicating a positive diagnosis of diseases. BMI, body mass index; TC, total cholesterol; HDL, high-density lipoprotein; BP blood pressure; HbA1c, haemoglobin A1c (glycated haemoglobin); SEP, socioeconomic position

Finally, Model 3 examined associations between childhood SEP and adult health, controlling for adult SEP (Figure 3). Compared with Model 2, the associations between childhood SEP and most health outcomes were attenuated. However, US SEP differences in current smoking were significant. Results were similar in sex-stratified models (Supplementary Material S10, available as Supplementary data at *IJE* online), except for persisting inequalities in HbA1c among British males.

Results using the full racially and ethnically diverse Add Health sample were similar (Supplementary Material S11, available as Supplementary data at *IJE* online), except for ‘any’ diabetes, which was higher in the USA. In general, the US-Britain gap was smaller for current smoking and self-rated health, but larger for obesity, blood pressure, cholesterol and HbA1c. Results were also similar when limiting obesity to measured height and weight in Add Health (Supplementary Material S12, available as Supplementary data at *IJE* online). The overall prevalence of obesity was higher in the USA, but conclusions were unchanged.

Finally, different operationalizations of heavy drinking (Supplementary Materials S13 and S14, available as Supplementary data at *IJE* online) show contrasting results when adopting US or British definitions of heavy drinking.

## Discussion

Our analyses identified a US mid-life health disadvantage similar to that observed at older ages.^1–4^ The health disadvantage is notable for obesity, hypertension and cholesterol, though British adults have greater probabilities of current smoking and worse self-rated health. Further, socioeconomic inequalities are typically wider in the USA, where health differences between the most and least advantaged are larger. For current smoking, and to a lesser extent diabetes, this is due to better health among socioeconomically advantaged US adults but worse health among socioeconomically disadvantaged adults compared with Britain. For hypertension and high cholesterol, socioeconomically advantaged US adults have comparable—or worse—health than socioeconomically disadvantaged British adults.

Our finding that hypertension and high cholesterol are more prevalent in the USA supports previous research documenting worse cardiometabolic health among older US adults.^2^^,^^4^ Whereas research on mid-life is more limited, our results mirror extant research on a US disadvantage for obesity and cardiometabolic health,^5^ but differ from work documenting worse hypertension and dyslipidaemia (high cholesterol) among English adults in mid-life.^10^ Moreover, the risk of diabetes in the USA was higher only when considering the full ethnically heterogeneous sample in Add Health; this differs from previous work documenting 2-fold greater diabetes prevalence among US older adults, even in a non-Hispanic White sample, suggesting a possible increase in diabetes risk among younger British cohorts.^10^ Nevertheless, we find substantial evidence of poor mid-life health in both cohorts, supporting prior literature on declines in mid-life health across multiple domains in both countries,^21–24^ further underscoring the importance of studying healthy ageing as a lifelong process.^7^^,^^25^

Previous work found the prevalence of risky behaviours, including smoking,^1^^,^^5^ to be more common among English adults despite their lower chronic disease risk—consistent with the findings in this study. The seemingly contradictory nature of the disconnect between health behaviours and outcomes reaffirms past work suggesting the US health disadvantage is attributable to a multitude of both individual-level mechanisms (e.g. diet, physical activity and other lifestyle factors) and broader, social determinants of health (e.g. structural and policy factors shaping economic and educational opportunities and rewards). The interplay of these mechanisms remains an important area of future research.

However, harmonizing self-reported measures remains a challenge in internationally comparative research, where the subjective nature of both interpretation and reporting can pose issues. For example, in the Supplementary materials (Supplementary Materials S13 and S14, available as Supplementary data at *IJE* online), we show how US-Britain differences in heavy drinking differ based on its operationalization, specifically the extent to which more or less conservative national guidelines capture different ends of distributions of drinking behaviours.

The different sociopolitical contexts between the USA and Britain might help explain why, for several outcomes, the most socioeconomically advantaged respondents in the USA have equal or worse health compared with the most disadvantaged in Britain. For example, the US and British health care systems differ substantially.^26^ Britain has the National Health Service, which is universally available and free at the point of access. In the USA, health care is largely privatized, and the associated costs are often high regardless of access. Past work has suggested that relatively ‘universal’ access to health care at older ages in the USA through Medicare helps explain its better international standing in mortality and morbidity for medically amenable causes of death.^27^ However, US-England comparisons of income gradients in health at older ages found no differences in England compared with a clear gradient in the USA, likely due to a more generous benefit system for older English adults where, below the median income, retirement benefits are largely consistent and unrelated to historical income.^3^

Given our results, it is likely that differences between the USA and Britain reflect broader inequalities affecting population health. Societies with higher levels of inequality typically have worse health across a range of metrics,^28^ with more unequal countries having steeper socioeconomic gradients and worse average health outcomes regardless of one’s SEP. From that perspective, the unique combination of high inequality and a weak welfare state in the USA may prove harmful for all groups throughout the life course.

## Strengths and limitations

This research uses data from two nationally representative cohort studies in Britain and the USA, exploring health differences on a range of outcomes, including biomarkers. BCS70 is representative of Britain at time of recruitment; however, most of the cohort is White, preventing adjustments for race/ethnicity in both studies. As such, we were limited in our ability to explore intersectional relationships among race/ethnicity, gender and SEP—particularly in the US context, where racial/ethnic groups do not enjoy the same health benefits of advantaged SEP, due to structural factors.^29^ Differences in the ways SEP confers health advantage or disadvantage across racial/ethnic groups between the two countries remains an important future consideration.

Our extensive harmonization of measures between the two cohorts included the development of novel weights in BCS70, allowing for comparative analyses that account for Add Health’s complex survey design. Despite efforts to address attrition through use of non-response weights, the derivation of weights was not identical.

There may also be residual differences in how variables were measured and understood, particularly for subjective measures such as smoking and self-rated health, where questions are asked and/or possibly interpreted differently. For example, in additional analyses, we find that different operationalizations of heavy drinking (Results Supplementary Material S7, available as Supplementary data at *IJE* online) yield different patterns of inequities between the two countries owing to the different units of measurement (i.e. drinks vs units) and applications of national drinking guidelines.

Despite comprehensive efforts to harmonize the two studies, we cannot fully address all differences between the two cohorts. Though the birth cohorts represented by BCS70 and Add Health are similar, there may nevertheless be cohort differences influencing our results. The timing of the ‘obesity epidemic’ in the USA and Britain is one such consideration.^30^ The US Add Health cohort may have experienced a longer duration of obesity throughout their lives, with greater ramifications across multiple mid-life health indicators.^31^ Although the British BCS70 cohort was also subject to rising obesity from early adolescence,^32^ their comparatively lower cumulative exposure may be observed in a lower cardiometabolic health burden. Whereas past work finds that obesity explains a small proportion of country differences,^4^ it is possible that the accumulated, cohort- and country-specific nature of exposures plays a distinct role.

## Conclusion

US adults in mid-life have worse cardiometabolic health than their British counterparts, with wider SEP inequalities across multiple health outcomes. For some cardiometabolic outcomes, even the most advantaged SEP groups in the USA have worse health than all groups in Britain. Critically, our work highlights the need for more efforts to harmonize international datasets at younger ages—and especially mid-life—to better understand the emergence of health inequalities throughout the life course and how this varies across countries.

## Ethics approval

Ethics approval was not sought for this analysis, which is analysis of publicly available secondary data. For BCS70, ethics approval is obtained from a National Health Service Research Ethics Committee in advance of each sweep of data collection. The Age 38 Survey was approved by Southampton & South West Hampshire Research Ethics Committee (08/H0504/144), the Age 42 Survey by London-Central Research Ethics Committee (11/LO/1560) and the Age 46 Survey by South East Coast—Brighton & Sussex (15/LO/1446). In addition, London-Central Research Ethics Committee have provided ethics approval for the ongoing activities of the study in between sweeps of data collection: keeping in touch and tracing study members; cleaning, documenting and providing access to the data for research; and linking data from administrative sources to survey data to increase the utility of the data for research (14/LO/0371). Add Health participants provided written informed consent for participation in all aspects of Add Health in accordance with the University of North Carolina School of Public Health Institutional Review Board guidelines that are based on the Code of Federal Regulations on the Protection of Human Subjects 45CFR46: [https://www.hhs.gov/ohrp/humansubjects/guidance/45cfr46.html].

## Supplementary Material

dyae127_Supplementary_Data

## Data Availability

All data in BCS70 are available through an end user licence through UKDS: [https://beta.ukdataservice.ac.uk/datacatalogue/series/series?id=200001]. Add Heath data can be accessed through a data application, see further details here: [https://addhealth.cpc.unc.edu/data/]. All code associated with the current analysis can be found in the following OSF repository: [https://osf.io/vkf2g/?view_only=89368400bf79432581a66fc681d914e7].
